# Effects of Maternal Fiber Intake on Intestinal Morphology, Bacterial Profile and Proteome of Newborns Using Pig as Model

**DOI:** 10.3390/nu13010042

**Published:** 2020-12-25

**Authors:** Ying He, Xie Peng, Yang Liu, Qing Wu, Qiang Zhou, Liang Hu, Zhengfeng Fang, Yan Lin, Shengyu Xu, Bin Feng, Jian Li, Yong Zhuo, De Wu, Lianqiang Che

**Affiliations:** 1 Key Laboratory for Animal Disease-Resistant Nutrition of the Ministry of Education of China, Institute of Animal Nutrition, Sichuan Agricultural University, Chengdu 611130, China; HedyYingHe@hotmail.com (Y.H.); pengxie2014@foxmail.com (X.P.); 18227585648@163.com (Y.L.); 13629039265@163.com (Q.W.); zq690096418@sina.com (Q.Z.); fangzhengfeng@hotmail.com (Z.F.); able588@163.com (Y.L.); shenyu_x@hotmail.com (S.X.); fengb123d@163.com (B.F.); lijian522@hotmail.com (J.L.); zhuoyong@sicau.edu.cn (Y.Z.); wude@sicau.edu.cn (D.W.); 2 College of Food Science, Sichuan Agricultural University, Ya’an 625014, China; huliangsau90@hotmail.com

**Keywords:** maternal fiber intake, intestinal morphology, bacterial profile, proteome, newborn piglets

## Abstract

Dietary fiber intake during pregnancy may improve offspring intestinal development. The aim of this study was to evaluate the effect of maternal high fiber intake during late gestation on intestinal morphology, microbiota, and intestinal proteome of newborn piglets. Sixteen sows were randomly allocated into two groups receiving the control diet (CD) and high-fiber diet (HFD) from day 90 of gestation to farrowing. Newborn piglets were selected from each litter, named as CON and Fiber group, respectively. Maternal high fiber intake did not markedly improve the birth weight, but increased the body length, the ileal crypt depth and colonic acetate level. In addition, maternal high fiber intake increased the α-diversity indices (Observed species, Simpson, and ACE), and the abundance of *Acidobacteria* and *Bacteroidetes* at phylum level, significantly increased the abundance of *Bradyrhizobium* and *Phyllobacterium* at genus level in the colon of newborn piglets. Moreover, maternal high fiber intake markedly altered the ileal proteome, increasing the abundances of proteins associated with oxidative status, energy metabolism, and immune and inflammatory responses, and decreasing abundances of proteins related to cellular apoptosis, cell structure, and motility. These findings indicated that maternal high fiber intake could alter intestinal morphology, along with the altered intestinal microbiota composition and proteome of offspring.

## 1. Introduction

The fetal intestine grows rapidly during the late gestation and then prepares for the transition from the uterine to outside environment during the perinatal period. It is well known that the main function of intestine is digestion of food and absorption of nutrients, which also plays a role in barrier and immune [1]. Pigs have a remarkable similarity to man in gut anatomy, physiology, as well as the prenatal and postnatal development of intestine. Therefore, the sow-piglet is a good model to study nutritional programming in human mother–infant [2]. Moreover, previous studies have proven that different nutrition plane and specific nutrition intake of sows during gestation or from gestation to lactation can affect intestinal development of offspring [3,4]. Specifically, these effects usually shown in altering intestinal morphology, microbiota, immune and inflammation, etc. [4,5].

As edible carbohydrate polymers, the effect of dietary fiber on intestinal function has been widely reported [6,7]. The beneficial effects of dietary fiber on intestinal function are ascribed to the modulating role on intestinal microbiota, fermentation metabolites (short-chain fatty acids, SCFAs), etc., in animal and human models [8,9,10]. Dietary fiber supplementation in animals can reduce intestinal inflammation, promoting intestinal antioxidant capacity and intestinal barrier function, etc. [11,12]. Some reports have proved that maternal fiber intake can significantly affect the development of offspring. A recent study demonstrated maternal low-fiber intake decreased the serum SCFAs levels in maternal and offspring mice and impaired the neurocognitive function of offspring mice [13]. Similarly, it has also been reported that maternal high fiber intake increased plasma SCFAs levels of maternal and offspring mice and regulated T cell differentiation in offspring mice [14]. Our study showed maternal fiber intake improved testis cell development and altered testicular proteome of offspring piglets [15]; we also found the composition of dietary fiber in pregnancy diet affect the intestinal microbiota composition of offspring piglets [16].

However, there are limited reports about the effect of maternal fiber intake on the intestinal development of fetus and underlying mechanism. To our knowledge, the effects of maternal fiber intake on fetal intestinal proteomic and bacterial profile have not been reported. Therefore, we hypothesized that maternal high fiber intake has beneficial effects on intestinal development, associating with altering intestinal microbiota and proteome.

## 2. Materials and Methods

All experimental protocols for the present study were approved by the Animal Care and Use Committee of Sichuan Agricultural University under permit number DKYB20131704 and carried out in accordance with the Ministry of Science and Technology in China’s Guide for the Care and Use of Laboratory Animals. The study was conducted in a commercial sow farm (Tianfu sow farm, which belongs to Sichuan Giastar Group, Chengdu, China).

### 2.1. Experimental Design, Diets and Management

**Figure 1:**
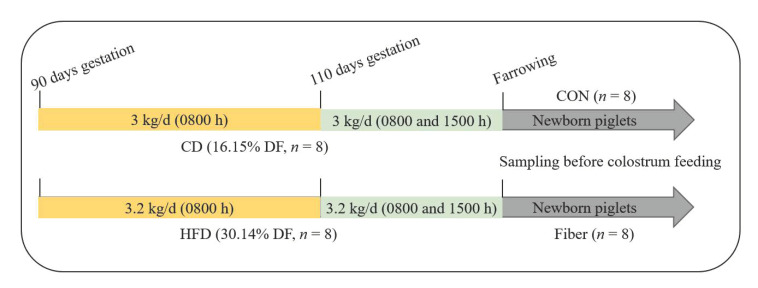
Schematic diagram of experimental design and diet. CD, Control diet; HFD, high dietary fiber diet; CON, Control group; Fiber, Fiber group.

A total of 16 Yorkshire sows with an average BW of 267.8 ± 2.8 kg and parity of 4–6 were selected to this study. Sixteen Yorkshire sows were randomly assigned to two groups (*n* = 8), Control diet (CD, 16.15% dietary fiber), and high dietary fiber diet (HFD, 30.14% dietary fiber) were fed, respectively. Dietary treatment was applied from day 90 of gestation to farrowing. Two diets were formulated based on corn-soybean meal to meet or exceed the recommendation of NRC (National Research Council, 2012) as shown in Table 1. The dietary fiber content in HFD was 1.8 times as many as the CD. Energy and protein intake were identical via adjusting feed intake (3.0 and 3.2 kg/d, respectively) from day 90 of gestation to farrowing. During day 90 to 110 of gestation, the diet was supplied once a day (0800 h). On day 111 of gestation, sows were moved to the farrowing room and feeding frequency turned to twice a day (0800 and 1500 h) until farrowing. Newborn piglet (average litter weight) was selected from each litter (*n* = 8), named as Control group (CON) and Fiber group (Fiber), respectively (Figure 1).

### 2.2. Sample Collection

Piglets birth weight and tissue samples were collected before sucking colostrum. After anesthetized (Zoletil 50; Virbac, Nice, France; 0.15 mL/kg), blood samples were collected into heparinized tubes (5 mL) by vena jugular. Plasma samples were then obtained by centrifuging the blood samples at 3000× *g* and 4 °C for 15 min and were stored at −20 °C until analysis. After blood sampling, all piglets were slaughtered. The weight of heart, liver, spleen, kidney, stomach, pancreas, large intestine, small intestine, and the length of body (crown to rump), head, and small intestine were measured. As our previous study [3], ileum tissue samples (about 2 cm in length) were preserved in 4% paraformaldehyde solution for histological measurements. Another portion of ileum tissue were freeze with liquid nitrogen and stored at −80 °C until assay. Fresh colonic digesta samples from middle of colon were collected into sterile tubes, and immediately frozen until assay.

### 2.3. Intestinal Morphology

The ileum tissue samples were fixed in 4% paraformaldehyde, then embedding in paraffin. Each ileum sample was cut into 5 μm thick slide by Leica RM2235 slicer, each sample had 5 slides and each slide had 3 sections, then stained with the periodic acid Schiff method (PAS staining). Images were taken under the microscope. Ten well-oriented villi-crypts units of each section (Image ProPlus 6.0) were measured and villi–crypt ratio (VCR) was calculated. Image ProPlus 6.0 was used to count the goblet cells and measure the tissue areas, then calculate the goblet cell density (number of cells/µm^2^), and goblet cells density were obtained from 3 sections by each small intestinal segment.

### 2.4. Plasma Biochemical Parameters

The plasma concentrations of alanine aminotransferase (ALT), aspartate aminotransferase (AST), gamma-glutamyltransferase (GGT), total bile acid (TBA), glucose (GLU), Urea, creatinine (CREA), and triglyceride (TG) were determined by automatic biochemical analyzer (Model 7020, Hitachi, Tokyo, Japan) through corresponding commercial kits (Sichuan Maker Biotechnology Inc., Chengdu, China).

### 2.5. The SCFAs Levels in Colonic Digesta

Accurately weighed 0.4 g of colonic digesta samples were added with 600 µL of ultra-pure water and methanol mixture (1:1), mixed thoroughly, and incubated at room temperature for 30 min, then centrifuged to collect supernatant, which was added with metaphosphoric acid solution and crotonic acid solution, and incubated at 4 °C for 30 min. Centrifuged to took 200 µL of supernatant and be added with 200 µL methanol. One microliter of supernatant was used for detecting levels of acetate, propionate, butyrate, isobutyrate, valerate, and isovalerate using gas chromatograph (Varian CP-3800, Palo Alto, CA, USA).

### 2.6. RNA Extraction and Real-Time Quantitative PCR

Total ileal RNA was extracted by RNAiso Plus (Takara) according to the instructions. Then reverse transcription of RNA and Real-time quantitative polymerase chain reaction (RT-qPCR) were performed by PrimeScript RT reagent Kit with gDNA Eraser (Takara) and TB Green Premix Ex Taq II (Takara), respectively. Both reactions were performed according to the instructions. All RT-qPCR target gene expression was normalized to the expression of housekeeping gene (GAPDH) and analyzed using the 2^−ΔΔCt^ method [17]. The PCR primers used in this study are listed in Table S1.

### 2.7. Bacterial Profile Analysis by 16S rRNA Gene Sequencing

Six colonic digesta samples were randomly selected from each group for bacterial profile analysis (Novogene Technology Co, Ltd., Beijing, China). Microbial DNA from colonic digesta samples was extracted by QIAamp DNA stool Mini Kit (Qiagen Gmb, Hilden, Germany). Then, 1% agarose gels were used to monitor DNA concentration and purity. According to the concentration, using sterile water dilute DNA to 1 ng/µL. High fidelity PCR was used for amplifying the bacterial 16S rDNA hypervariable V3-V4 region with the primers. Mixture PCR products was purified with Gene JETTM Gel Extraction Kit (Thermo Scientific, Waltham, MA, USA).

Sequencing libraries were generated using Ion Plus Fragment Library Kit 48 rxns (Thermo Scientific). The library quality was assessed on the Qubit@ 2.0 Fluorometer (Thermo Scientific). High-throughput sequencing was performed on an Ion S5TMXL platform and 250 bp single-end reads were generated after the library was quantified. Followed by data split and filtration (http://cutadapt.readthedocs.io/en/stable/) and removed Chimera to obtain clean reads. Sequences analysis were performed by Uparse software (Uparse v7.0.1001, http://drive5.com/uparse/, Robert Edgar, CA, USA) to obtain operational taxonomic units (OTUs) and species annotation were performed by Silva Database (https://www.arb-silva.de/). All the results were based on sequenced reads and OTUs. The relative abundances of OTUs were detected at different taxonomic levels, α-diversity indices, and Principal Coordinate Analysis (PCoA) analysis were calculated with QIIME (Version1.7.0), PCoA analysis was conducted based on the Weighted Unifrac distance.

### 2.8. iTRAQ Quantitative Proteomic Analysis

#### 2.8.1. Protein Preparation

Four ileum samples were randomly selected from each group and homogenized in extraction SDT buffer (4% *w*/*v* SDS, 1 mM DTT, 150 mM Tris-HCl, pH 8.0). The homogenate was sonicated and then boiled for 15 min, collecting the supernatant after centrifuged at 14,000× *g* for 40 min, and filtered it with 0.22 µm filters. BCA method was used for protein quantification. Then 5X loading buffer was added in protein samples (20 μg) and boiled for 5 min. Finally, the samples were separated by electrophoresis on 12.5% SDS-PAGE gels and stained with Coomassie Brilliant Blue to confirm parallelisms among samples.

#### 2.8.2. Protein Digestion and iTRAQ Labeling

Protein digestion was performed according to the FASP procedure described as before [18]. Then the 100 μg of peptide mixture was labelled with iTRAQ (isobaric tags for relative and absolute quantification) reagent according to the manufacturer’s instructions (Applied Biosystems, Carlsbad, CA, USA).

#### 2.8.3. Peptide Fractionation with Strong Cation Exchange (SCX) Chromatography

The iTRAQ-labeled peptides were fractionated by SCX chromatography using the AKTA Purifier system (GE Healthcare, Chicago, IL, USA). The dried peptide mixture was reconstituted and acidified with 2 mL buffer A (10 mM KH2PO4 in 25% of acetonitrile, pH 3.0) and loaded onto a Poly SULFOETHYL 4.6 × 100 mm column (5 µm, 200 Å, PolyLC Inc, Columbia, MD, USA). The elution was monitored by absorbance at 214 nm, and the fractions were collected every 1 min. The collected fractions were desalted on C18 Cartridges (Empore^TM^ SPE Cartridges C18 (standard density), bed I.D.7 mm, volume 3 mL, Sigma, St. Louis, Mo, USA) and concentrated by vacuum centrifugation.

#### 2.8.4. LC-MS/MS Analysis

Each fraction was injected for nanoLC-MS/MS analysis. The peptide mixture (5 μg) was loaded onto a C18-reversed-phase column (Thermo Scientific Easy Column, 10 cm long, 75 μm inner diameter, 3 μm resin) in buffer A (0.1% formic acid) and separated with a linear gradient of buffer B (84% acetonitrile and 0.1% formic acid) at a flow rate of 300 nL/min controlled by IntelliFlow technology over 140 min. MS data was acquired using a data-dependent top 10 method dynamically choosing the most abundant precursor ions from the survey scan (300–1800 *m*/*z*) for HCD fragmentation. Automatic gain control (AGC) target was set to 3 × 106, and maximum inject time to 10 ms. Dynamic exclusion duration was 40.0 s. Survey scans were acquired at a resolution of 70,000 at *m*/*z* 200 and resolution for HCD spectra was set to 17,500 at *m*/*z* 200, and isolation width was 2 *m*/*z*. Normalized collision energy was 30 eV and the underfill ratio, which specifies the minimum percentage of the target value likely to be reached at maximum fill time, was defined as 0.1%. The instrument was run with peptide recognition mode enabled.

#### 2.8.5. Sequence Database Search and Data Analysis

The MS/MS spectra were searched using MASCOT engine (Matrix Science, London, UK; version 2.2) embedded into Proteome Discoverer 1.4 (Thermo Electron, San Jose, CA, USA) against the Uniprot (uniprot_Sus_scrofa_50331_20190305). For protein identification, the following options were used: peptide mass tolerance = 20 ppm, MS/MS tolerance = 0.1 Da, enzyme = trypsin, missed cleavage = 2, fixed modification: Carbamidomethyl (C), iTRAQ 8plex(K), iTRAQ 8plex(N-term), variable modification: Oxidation (M), false discovery rate (FDR) ≤ 0.01.

#### 2.8.6. Bioinformatic Analysis

The protein sequences of differentially expressed proteins were in batches retrieved from the UniProtKB database (Release 2019_03) in FASTA format. In this work, Blast2GO (version 3.3.5) was used for GO mapping and annotation (27, Götz et al., 2008). The GO annotation results were plotted by R scripts. The FASTA protein sequences of differentially changed proteins were blasted against the online Kyoto Encyclopedia of Genes and Genomes (KEGG) database (http://geneontology.org/) to retrieve their KOs and were subsequently mapped to pathways in KEGG. GO enrichment on three ontologies (biological process, molecular function, and cellular component) and KEGG pathway enrichment analyses were applied based on the Fisher’s exact test. The Benjamini–Hochberg correction for multiple testing was further applied to adjust the derived *p*-values. Only functional categories and pathways with *p*-values under a threshold of 0.05 were considered as significant.

### 2.9. Correlation Analysis of the Microbiomic and Proteomic Data

Correlation analysis was performed by microbiomic and proteomic data from the corresponding four piglets. Spearman correlation coefficients were calculated between the colonic bacterial taxa data and the abundances of several proteins in ileum, a value of *p* < 0.05 was considered significant correlation.

### 2.10. Statistical Analysis

The individual piglet was used as the experimental unit for all response variables in the model, which included maternal diet (CD or HFD) as the main effect. The data shown in the study were expressed as mean ± standard error of the mean (SEM). Birth weights and organ index, intestinal morphology, plasma biochemical parameters, microbial alpha diversity indices, the SCFAs levels in colonic digesta, and gene expression in the ileum were analyzed by an independent-samples *t*-test. For analysis of intestinal microbiota, data of relative abundance at phylum and genus levels were analyzed by nonparametric Mann–Whitney U test. Statistical significance was assessed by SPSS 22.0 (IBM SPSS Company, Chicago, IL, USA), a value of *p* < 0.05 was considered statistically significant, whereas 0.05 ≤ *p* < 0.10 was considered a tendency.

## 3. Results

### 3.1. Birth Weights and Organ Index

Maternal high fiber intake had significantly increased body length and head length of piglets (*p* < 0.05), but not other organ indexes, relative to CON (Table 2).

### 3.2. Intestinal Morphology and Goblet Cell Density

**Figure 2:**
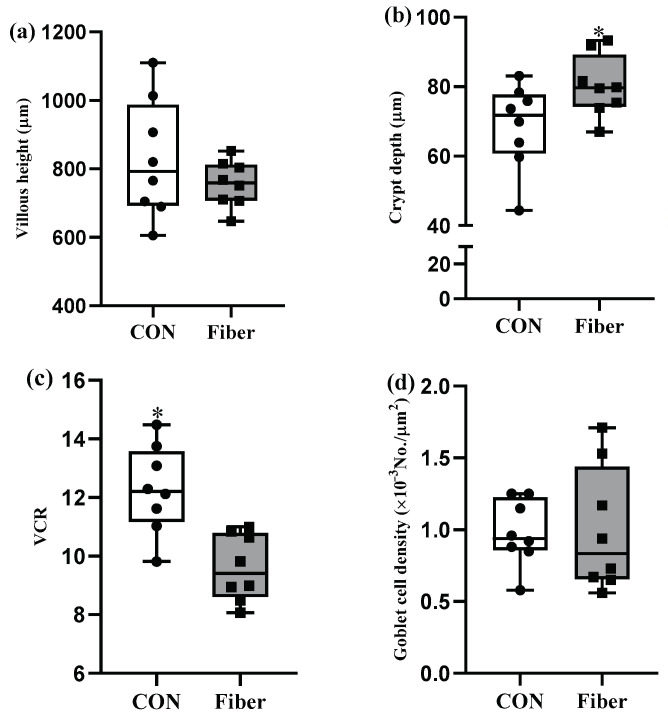
The effects of maternal fiber intake on ileal histomorphology and number of goblet cells of newborn piglets. (**a**), Villous height; (**b**), Crypt depth; (**c**), Ratio of villous height to crypt depth (VCR); (**d**), Goblet cell density of newborn piglets. Values are presented as box-and-whisker plots, * means *p* < 0.05.

Maternal high fiber intake had markedly increased crypt depth of ileum in newborn piglets (Figure 2b, *p* < 0.05), but no differences were observed for the villous height (Figure 2a) and goblet cell density (Figure 2d). Accordingly, maternal high fiber intake markedly decreased ratio of villous height to crypt depth (VCR) relative to CON group (*p* < 0.05, Figure 2c).

### 3.3. Plasma Biochemical Parameters

Maternal high fiber intake had markedly increased concentration of CREA in plasma of piglets (*p* < 0.05), but not other biochemical parameters, relative to CON (Table 3).

### 3.4. The SCFAs Levels in Colonic Digesta

**Figure 3:**
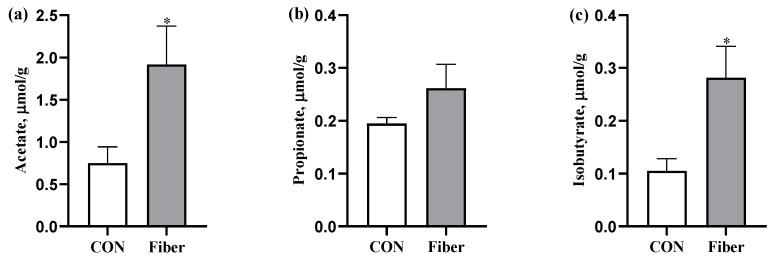
The effects of maternal fiber intake on short-chain fatty acids (SCFAs) levels in colonic digesta of newborn piglets. (**a**), Acetate; (**b**) Propionate; (**c**) Isobutyrate in colonic digesta. Values are presented as mean ± standard error (*n* = 8), * means *p* < 0.05.

Maternal high fiber intake had markedly increased levels of acetate and isobutyrate in colonic digesta of newborn piglets (*p* < 0.05), but no significant difference was observed for propionate (Figure 3).

### 3.5. Bacterial Profile Analysis of Colonic Digesta

**Figure 4:**
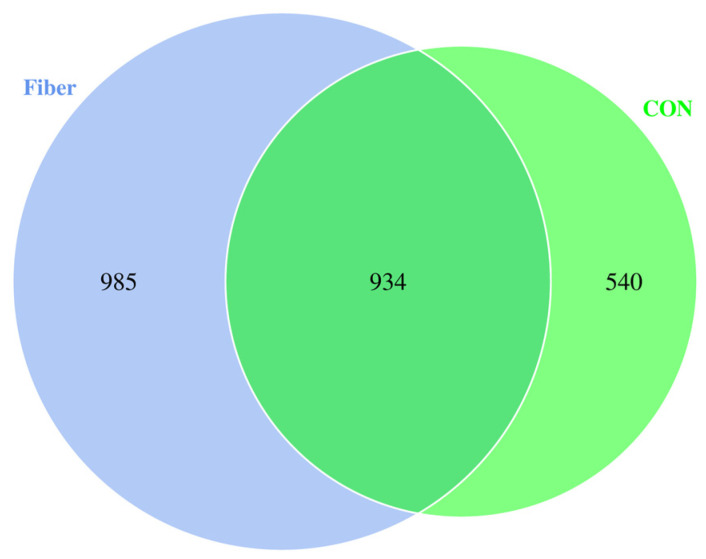
Venn diagram showing the unique and shared operational taxonomic units (OTUs) in the two groups (*n* = 6 for each group).

After OTUs were assigned and chimeras were removed, sequencing of 12 colonic digesta samples generated 804,732 quality sequences with an average of 67,061 ± 4211 (mean ± standard error) sequences per sample. A total of 7127 OTUs were identified and each sample had 593 ± 52 (mean ± standard error) OTUs on average. Wherein 934 OTUs were common in two groups, 540 and 985 unique OTUs were identified in CON and Fiber groups (Figure 4). The mean of Good’s coverage for all samples was high (>98%), indicating that the majority of the microbial phylotypes in the colonic digesta samples were covered.

**Figure 5:**
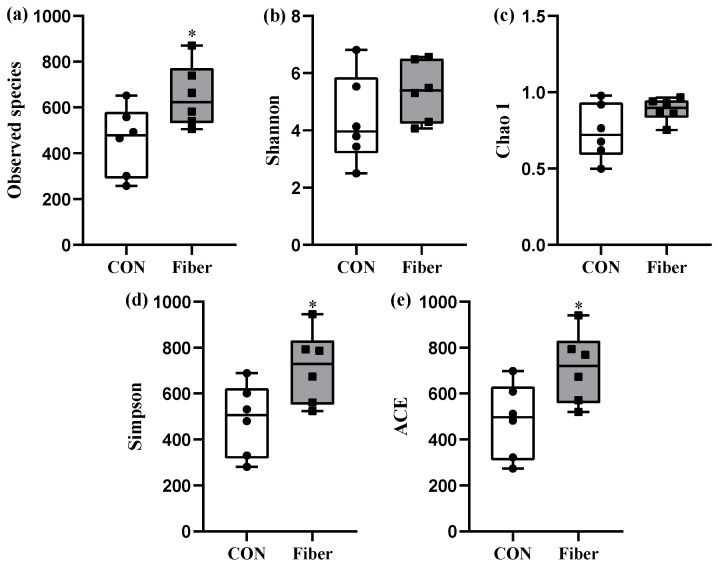
Differences in bacterial community diversity and richness in the two groups. (**a**), Observed species; (**b**), Shannon; (**c**), Chao 1; (**d**), Simpson; (**e**) ACE. Values are presented as box-and-whisker plots, * means *p* < 0.05.

The Observed species, Shannon, Simpson, Chao1, and ACE indices can represent alpha diversity for two groups, maternal fiber intake had significantly increased both richness indices (Observed species and ACE) and diversity index Simpson of newborn piglets (*p* < 0.05, Figure 5). Beta diversity was illustrated via PCoA (Principal Co-ordinates) analysis, there was no significant difference in microbial community structure between the two groups (Figure S1).

**Figure 6:**
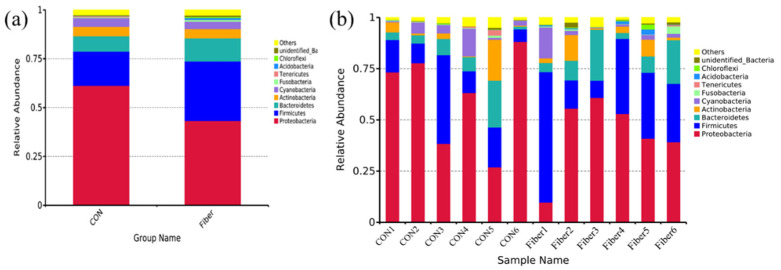
Top 10 bacterial phyla of microbial community in colonic digesta of newborn piglets between two groups (**a**) and each sample (**b**).

In terms of bacterial composition at phylum level, five phyla, such as *Proteobacteria*, *Firmicutes*, *Bacteroidetes*, *Actinobacteria*, and *Cyanobacteria* had a relative abundance greater than 1.0% in at least one experimental group (Figure 6). Of these, *Proteobacteria* predominated in all samples, with a relative abundance of 9–88%, followed by *Firmicutes* (6–63%), *Bacteroidetes* (0.27–24%), and *Actinobacteria* (0.03–13%). Maternal high fiber intake had significantly increased abundance of *Acidobacteria* at phylum level of newborn piglets (*p* < 0.05) and tended to increase abundance of *Bacteroidetes* (Table 4, *p* = 0.065).

**Figure 7:**
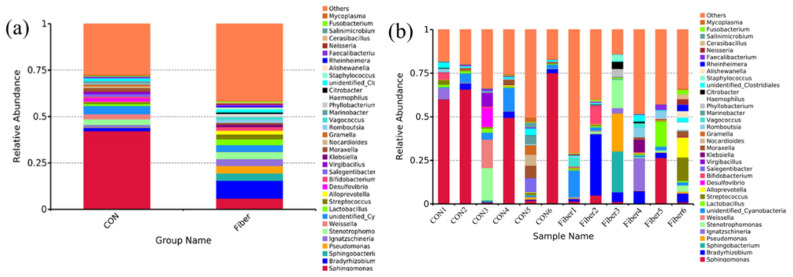
Top 35 bacterial genera of microbial community in colonic digesta of newborn piglets between two groups (**a**) and each sample (**b**).

Among all samples, the top 35 bacterial genera were identified (Figure 7). Nineteen genera had relative abundance greater than 1.0% in at least one experimental group. Of these, *Sphingomonas* predominated in all samples. Maternal high fiber intake had markedly increased relative abundances of *Bradyrhizobium* and *Phyllobacterium* of newborn piglets (Table S2, *p* < 0.05).

### 3.6. Identification and Quantification of Differentially Abundant Proteins

#### 3.6.1. Protein Profiling

In this study, using iTRAQ analysis, a total of 6418 proteins (Table S3) were identified. Using a 1.2-fold increase or decrease in protein abundance as a benchmark for physiologically significant change, a total of 206 different abundance proteins (DAPs) were reliably quantified using iTRAQ analysis. Among those, 70 proteins were up-regulated, while 136 proteins were down-regulated (Table S4).

**Figure 8:**
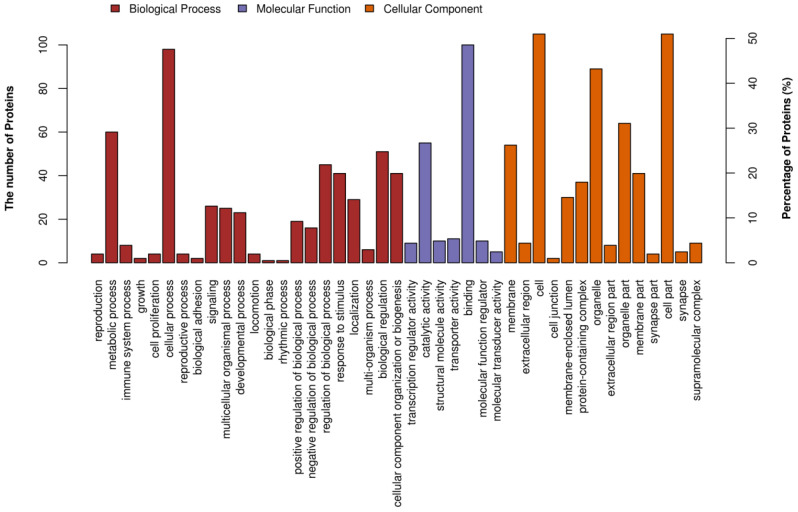
Gene ontology (GO) analysis of total proteins based on the categories cellular component, biological process and molecular function.

The gene ontology (GO) analysis of total proteins produced 3 major functional categories: biological process, cellular component, and molecular function (Figure 8). Among the biological processes, “cellular process” was the most commonly represented, followed by “metabolic process” and “biological regulation”. In the category of molecular function, the significant proportion of clusters were assigned to “binding” and “catalytic activity”. Proteins involved in the “cell part”, “organelle”, “cell”, “organelles part”, and “membrane” groups were notably represented in the cellular component category.

#### 3.6.2. iTRAQ Quantification and Database Enrichment Analyses

**Figure 9:**
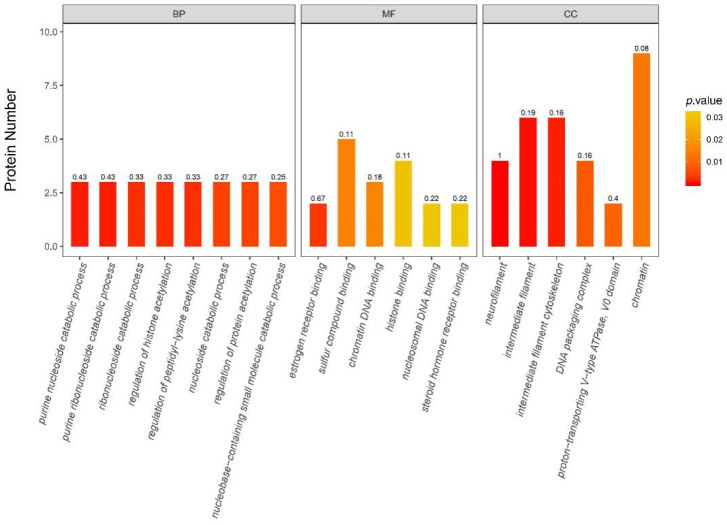
GO enrichment analyses of all differentially abundant proteins (Top 20 gene ontology clusters, *p*-value of Fisher’s exact test < 0.05). BP, biological process; MF, molecular function; CC, cellular component.

By performing the GO enrichment analysis, a total of 203 GO clusters were found to significantly change after maternal high-fiber intake (*p* < 0.05). In the top 20 GO clusters (Figure 9), eight GO clusters belonged to the biological process, which is mainly related to catabolic process of purine nucleoside, purine ribonucleoside, nucleoside, ribonucleoside, nucleobase-containing small molecule, and regulation of histone, peptidyl-lysine acetylation, protein regulation of acetylation. Five GO clusters belonged to the molecular function, which is mainly related to estrogen receptor binding, sulfur compound binding, chromatin DNA binding, histone binding, nucleosomal DNA binding, and steroid hormone receptor binding. Among the cellular component, neurofilament, intermediate filament, intermediate filament cytoskeleton, DNA packaging complex, proton-transporting V-type ATPase, VO domain and chromatin were significantly changed.

**Figure 10:**
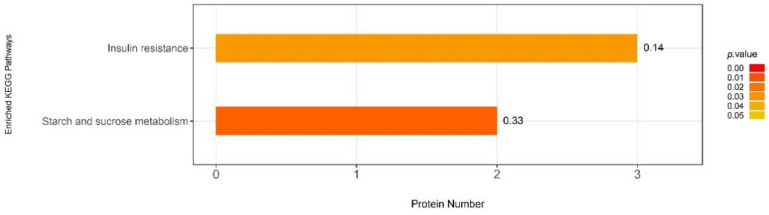
KEGG enrichment analyses of all differentially abundant proteins (*p*-value of Fisher’s exact test < 0.05).

By performing the KEGG pathway enrichment analyses, a total of two pathways that changed significantly (*p* < 0.05) after maternal high fiber intake (Figure 10). Among them, the insulin resistance pathway was significantly enriched.

#### 3.6.3. Functions of the Selected DAPs

There are 206 DAPs between CON and high fiber groups, on the basis of their biological functions, proteins are mainly classified into five groups: (1) cellular apoptosis; (2) oxidative status; (3) energy metabolism; (4) immune and inflammatory responses; and (5) cell structure and motility. Biochemical information about these proteins was summarized in Table 5.

#### 3.6.4. Validation of DAPs by RT-PCR

**Figure 11:**
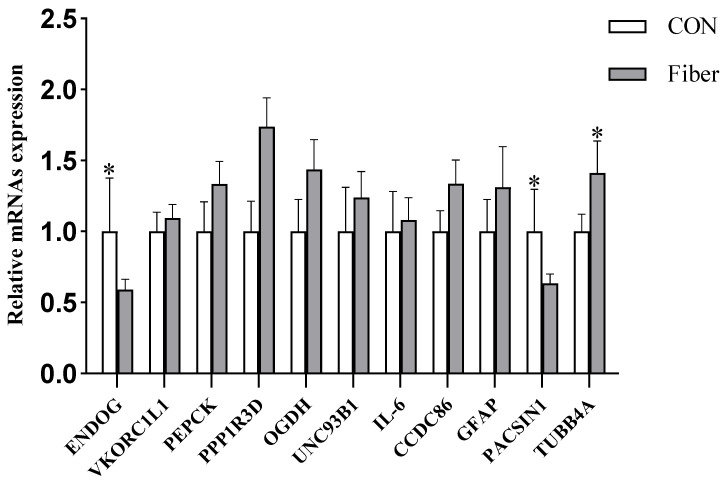
Validation of DAPs by RT-PCR, the relative expression of mRNA. Values are means, with their standard errors represented by vertical bars, * means *p* < 0.05. ENDOG, endonuclease G; VKORC1L1, vitamin K epoxide reductase complex subunit 1 like 1; PEPCK, phosphoenolpyruvate carboxykinase 2; PPP1R3D, protein phosphatase 1 regulatory subunit 3D; OGDH, oxoglutarate dehydrogenase; UNC93B1, unc-93 homolog B1; IL6, interleukin 6; CCDC86, coiled-coil domain containing 86; GFAP, glial fibrillary acidic protein; PACSIN1, protein kinase C and casein kinase substrate in neurons 1; TUBB4A, tubulin beta 4A class Iv.

To further validate the iTRAQ results, we selected 11 genes to perform RT-PCR verification. Maternal high fiber intake significantly reduced the mRNA expressions of ENDOG and PASCIN1 (*p* < 0.05), but significantly increased the mRNA expressions of TUBB4A (*p* < 0.05), and there was no significant effect on other gene expressions (Figure 11). The protein levels of ENDOG and PASCIN1 are consistent with their mRNA expression.

### 3.7. Correlation Analysis of the Microbiomic and Proteomic Data

**Figure 12:**
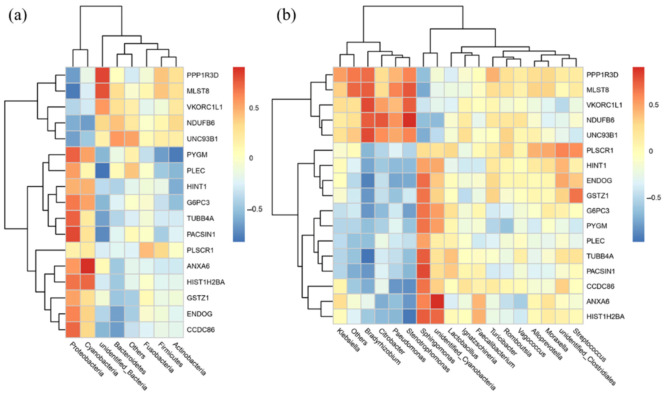
Heatmap of correlation of the ileal proteomics and colonic bacterial taxa at phylum level (**a**) and genus level (**b**). Correlation coefficients were colored according to the scale listed on the right.

Correlation between the abundance of colonic bacterial taxa and proteomic data was conducted by Spearman Correlation analysis (Figure 12). Between two groups of newborns, multiple bacterial taxa were correlated with the protein expressions. At phylum, *Proteobacteria* was correlated with CCDC86, TUBB4A, PASCIN1, MLST8, and PYGM, while *Actinobacteria* was correlated with PYGM. At genus level, *Sphingomonas* was correlated with HIST1H2BA, TUBB4A, PACSIN1, UNC93B1, and MLST8. Of note, both *Bradyrhizobium* and *Stenotrophomonas* were associated with VKORC1L1, NDUFB6, MLST8, ENDOG, G6PC3, and CCDC86. In addition, *Pseudomonas* was associated with ENDOG, CCDC86, while *Citrobacter* was correlated with NDUFB6 and ANXA6.

## 4. Discussion

In this study, results showed that maternal high fiber intake during late gestation altered the body length, intestinal morphology and microbiota, as well as the proteome of ileum in newborn piglets. The body length (crown to rump) of intrauterine growth restriction (IUGR) is less than normal littermates [19], and it has been proposed as one of the predictors for postnatal performance [20]. The increased body length of newborns in fiber group indicate that maternal fiber intake affects prenatal and postnatal development.

Villous height is regarded as a sign of surface area of nutrient absorption, and crypt depth can reflect the epithelial cell proliferation, which could be deeper if there is higher epithelial cell turnover [21]. Late gestation is the rapid development period for fetal intestine [22]. Using pig as model showed that fetuses at day 110 of gestation had greater crypt depth than that of fetuses at day 90 of gestation, suggesting fetal crypt depth is positively correlated with intestinal maturation [23].

In this study, therefore, the increased crypt depth in newborns from fiber group suggested maternal high fiber intake may enhance intestinal maturation. Furthermore, the intestinal microbiota plays a vital of important role in postnatal intestinal function, immune system and body metabolism [24], and maternal fiber intake during gestation influences the composition of intestinal microbiota in 14-day old piglets [25]. Using the 16S rRNA gene sequencing, in this study, maternal high fiber intake markedly increased the α-diversity indexes (Observed species, Simpson and ACE.) of newborn piglets. Observed species is the amount of unique OTUs found in each sample, Simpson, and ACE were used to estimate the species diversity and richness of microbiome, respectively [26]. Intestinal microbiota with high diversity could be more stable and healthier than low diversity, while low diversity is always associated with metabolic syndrome and inflammation [27,28]. At phylum level, *Proteobacteria*, *Firmicutes*, *Bacteroidetes*, and *Actinobacteria* were the most predominant phyla in the colon of newborn piglets, which were consistent with those previously found in the colon of newborn piglets [16]. Maternal high fiber intake significantly increased the abundance of *Acidobacteria* and showed a tendency to increase the abundance of *Bacteroidetes*. Some genera or species of *Acidobacteria* were associated with carbohydrate metabolism, nitrogen metabolism, exopolysaccharide production and transporter functions [29]. Moreover, feeding lactobacillus plantarum cell-free extract to whiteleg shrimp penaeus vannamei had significantly increased the abundance of *Acidobacteria* in intestine [30]. Dietary fiber intake could increase the abundance of *Bacteroidetes* in feces of rat and pig [31,32]. Moreover, a recent study demonstrated that a soluble fiber diet increased some species of *Bacteroidetes* and IgA production in the intestine of mice [33]. In this study, interestingly, maternal high fiber intake had increased the abundance of *Bacteroidetes* in newborn piglets. At the genus level, moreover, maternal high fiber intake markedly increased the relative abundances of *Bradyrhizobium* and *Phyllobacterium* in newborn piglets. The bacterial colonization of newborns may occur prenatally, which originating from the maternal gut, vagina, amniotic fluid, and placenta, the bacterial profile is mainly influenced by diet, antibiotic exposure, stress, and health status of dam [34,35]. In this study, therefore, we could not exclude the altered intestinal microbiota of newborns may be resulting from the altered maternal microbes by dietary fiber. In this study, we further detected the levels of SCFAs in colonic digesta of newborns, and found the increased level of acetate. Similarly, maternal dietary fiber supplementation significantly increased the level of acetate in plasma and feces of suckling piglets [25]. Moreover, maternal transfer of acetate to offspring had been proposed to be crucial factor in reducing the incidence of asthma [36] and enhancing T cell development in offspring [37]. In our study, therefore, the increased acetate in colonic digesta of newborns may be a crucial factor to affect intestinal function.

To further study the intestinal function responses to maternal fiber intake, the iTRAQ approach was used for quantifying changes of proteins expressions of newborn intestine. Compared with the traditional 2D-DIGE approach, iTRAQ approach can be more precise and identify more proteins [38]. Previous studies have shown that maternal high-fat intake during gestation significantly altered the intestinal proteome of neonatal mice [39], and our recent study showed that maternal high fiber intake significantly altered the testicular proteome of newborn pigs [15]. In the present study, likewise, maternal high fiber intake altered the proteome of ileum, relating to cellular apoptosis, oxidative statue, energy metabolism, immune and inflammatory responses, cell structure, and motility.

### 4.1. Cellular Apoptosis

The poor intestinal morphology is generally accompanied with the increased intestinal epithelial cells apoptosis [40]. In this study, we found the maternal fiber intake decreased the abundances of Phospholipid scramblase (PLSCRs), Endonuclease G (EndoG), Annexin (AnxA6), Apoptosis antagonizing transcription factor (AATF), and Histidine triad nucleotide binding protein 1 (HINT1), which participate in cellular apoptosis. PLSCRs are a family of transmembrane proteins inducing the release of mitochondrial cytochrome C for cellular apoptosis [41]. EndoG is a nuclear-encoded apoptosis endonuclease, once released from mitochondria, it could cause an DNA fragmentation and chromatin condensation [42]. AnxA6 is a Ca^2+^ dependent membrane-binding protein, and lidocaine-treated PC12 cells significantly increased cell apoptosis and gene expression of ANXA6 [43]. As a tumor suppressor, moreover, the overexpression of HINT1 up-regulated expressions of p53 and proapoptotic factor Bax, down-regulated the apoptosis inhibitor Bcl-2 to trigger apoptosis in SW480 and MCF7 cancer cells [44]. Furthermore, the abnormal intestinal morphology was accompanied by increased mRNA and protein expression of Bax in rat acute biliary infection model [45]. Therefore, maternal high fiber intake decreased most of protein abundances related to cellular apoptosis and may be beneficial for intestinal morphology.

### 4.2. Oxidative Status

Maternal high fiber intake markedly increased the abundances of Vitamin K epoxide reductase complex, subunit 1-like 1 (VKORC1L1), but deceased the Glutathione S-transferase zeta 1 (GSTZ1). VKORC1L1 is the key enzyme of the classical vitamin K cycle associating with oxidative stress, and is responsible for antioxidant capability by limiting intra-cellular ROS and adjusting Vitamin K to increase cell survival in human embryonic kidney cells [46]. Similarly, the mRNA expression and activity of VKORC1L1 were increased in hydrogen peroxide-treated HEK cells, and VKORC1L1 knock-down decreased cell viability [47]. As an antioxidant enzyme, moreover, the GSTZ1 expression were significantly increased in LPS-treated HUVECs, along with the increased ROS level [48]. In both mammals and rodents, intestinal morphology is closely related to oxidative stress, which can cause intestinal dysfunction [49,50]. These results indicate that maternal high fiber intake may have effects on improving oxidative status of newborns.

### 4.3. Energy Metabolism

In human-being, the supplementation of fiber could promote SCFAs production and energy metabolism [51]. In this study, we found maternal high fiber intake tended to increase the abundance of colonic *Bacteroidetes*, which plays an important role in maintaining energy homeostasis in animal model and human-being, and the abundance of intestinal *Bacteroidetes* is related to insulin resistance in mice and human [52,53]. By performing the KEGG pathway enrichment analyses, the insulin resistance pathway was significantly enriched. Three DAPs were associated with this pathway. Maternal high fiber intake markedly increased the expression of Protein phosphatase 1 regulatory subunit (PPP1R3D), but decreased the expression of lucose-6-phosphatase (G6PC3) and alpha-1,4 glucan phosphorylase (PYGM). Some studies suggested that dietary fiber supplementation reduced blood sugar and improved insulin sensitivity via decreasing the expression of G6PC3 in the liver of rodent animals [54,55]. Moreover, fiber metabolites SCFAs down-regulated G6PC3 expression of liver by activating AMPK, thereby inhibiting gluconeogenic glucose production [56]. As a glycogen targeting subunit of protein phosphatase type 1 (PP1), PPP1R3D participate in glycogen synthesis by joining with PP1 catalytic subunit and PP1 glycogen substrates [57]. Moreover, PPP1R3D regulated the glucose-induced inactivation of AMPK through binding to AMPKβ1 [58]. In this study, therefore, maternal high fiber intake may promote glycogen synthesis, while inhibit glycogen catabolism and glucose production. This means that maternal high fiber intake may alleviate insulin resistance and has a positive effect on intestinal glucose homeostasis, which had been recently proposed as an important effects in the energy homeostasis of body [59]. In addition, NDUFB6 is a subunit of the ubiquinone oxidoreductase complex I, which plays an important role in transferring electrons from NADH to the respiratory chain and ATP production [60]. In the present study, maternal high fiber intake increased the expression of NDUFB6, suggesting that maternal fiber intake may improve electron transfer and production of ATP.

### 4.4. Immune and Inflammatory Responses

In the present study, maternal high fiber intake significantly reduced the expressions of Coiled-coil domain containing 86 (CCDC86) and Histone H2B (HIST1H2BA), but increased the expressions of Unc-93 homolog B1 (UNC93B1). CCDC86 is a downstream molecule of IL-3, which can be induced by IL-3 in hematopoietic cell line [61]. As one of the histones, histone H2B can be harmful after entering to the extracellular space, and the administration of exogenous histones to animals leaded to systemic inflammatory and toxic responses, through activating Toll-like receptors and inflammasome pathways, extracellular histones may serve as inflammatory biomarkers in human diseases [62]. UNC93B1 is an endoplasmic reticulum resident transmembrane protein regulating the nucleic acid sensing Toll-like receptors [63]. Previous study has shown that human patients lacking functional UNC93B1 could develop Herpes simplex virus type 1 encephalitis [64]. Taken together, these results indicate that maternal high fiber intake may affect the cellular immune response to potential immunological stimulants in newborns.

### 4.5. Cell Structure and Motility

The expressions of Tubulin beta chain (TUBB4A), Plectin (PLEC), Protein kinase C, and casein kinase substrate in neurons 1 (PACSIN1) and Glial fibrillary acidic protein (GFAP) were significantly reduced, while the expression of LST8 homolog (MLST8) was increased in ileum of piglets from dams receiving high fiber intake. TUBB4A participate in cytoskeleton construction via organizing microtubules [65]. PLEC is a member of Plakin family proteins and widely distributed in gastrointestinal tract, skin, muscles etc. [66]. In addition, PLEC plays an important role in linking cytoskeleton components [67]. PACSIN1 is a cytoplasmic phosphoprotein, and serves as an essential function in actin reorganization, vesicle transport, and microtubule activities [68]. As a type III intermediate filament family, GFAP plays an important role in maintaining cytoskeleton and scaffold integrity [69]. Moreover, MLST8 is one of mTORC2 associated protein and function for controlling the actin cytoskeleton [70]. In short, these findings indicate that maternal fiber intake may influence intestinal cell structure and motility of newborns.

In this study, maternal high fiber intake altered the gut microbiome and proteome of newborns, and the correlation analysis of microbiomic and proteomic data showed that the pathogenic bacteria *Proteobacteria* and *Sphingomonas* [71] were positively correlated with protein expressions of CCDC86, HIST1H2BA, TUBB4A, and PASIN1 that are related to inflammation and cell structure [61,62,65,68]. *Proteobacteria* and *Actinobacteria* were positively correlated with PYGM, while *Bradyrhizobium* and *Stenotrophomonas* were negatively correlated with G6PC3, but positively correlated with NDUFB6, that are all associated with host energy homeostasis [56,60,72]. These results further indicate the altered intestinal development by maternal fiber intake may be related to the altered gut microbiome.

## 5. Conclusions

Our results indicated that maternal high fiber intake during late gestation had no significant effect on the birth weight of newborn piglets, but increased body length, ileal crypt depth, and colonic acetate level and altered microbiota composition of colonic digesta and ileal proteome of newborns. Particularly, maternal high fiber intake increased levels of proteins associating with oxidative status, energy metabolism, immune and inflammatory responses, as well as the decreased levels of proteins related to cellular apoptosis, cell structure and motility. The correlation analysis revealed the potential role of gut microbiome on the effect of maternal fiber intake on offspring intestinal development.

## Figures and Tables

**Table 1:** Ingredients and nutrient levels of experimental diets.

	CD	HFD
Corn (crude protein 8.2%)	73.50	49.83
Soybean meal (crude protein 46%)	16.80	11.30
Wheat bran	2.90	12.00
Soybean hull	2.90	12.00
Sugar beet pulp	0.00	11.40
L-Lysine-HCl (70%)	0.02	0.00
DL-Methionine (98.5%)	0.01	0.04
L-Threonine (98.5%)	0.00	0.04
CaCO3	1.08	0.67
CaHPO4	1.70	1.63
Sodium chloride	0.40	0.40
Choline chloride (50%)	0.14	0.14
Vitamin and mineral premix ^1^	0.55	0.55
Total	100.00	100.00
**Nutrient levels**		
Digestible energy, MJ/kg	13.31	12.47
Crude protein, %	14.23	13.29
Ether extract, %	3.07	2.82
Crude fiber, %	2.87	8.05
Neutral detergent fiber, %	10.11	21.93
Dietary fiber, %	16.15	30.14
Available P, %	0.46	0.43
Lysine, %	0.57	0.53
Methionine, %	0.22	0.21
Threonine, %	0.47	0.44
Tryptophan, %	0.15	0.18

^1^ Provided per kg of diet: Zn (as zinc sulfate), 100 mg; Cu (as copper sulfate), 6 mg; Fe (as ferrous sulfate), 100 mg; Mn (as manganese sulfate), 10 mg; I (as potassium iodide), 0.14 mg; Se (as sodium selenite), 0.25 mg; Cr (as chromium picolinate), 0.3 mg; VA, 14 mg; VB6, 14 mg; VE, 30 mg; VC, 100 mg; biotin, 0.1 mg; folic acid, 2.5 mg; carnitine, 46 mg.

**Table 2:** Organ index of newborn piglets.

	CON	Fiber	*p*-Value
Birth weight, kg	1.29 ± 0.03	1.28 ± 0.04	0.834
Body length, cm	35.9 ± 0.7	37.9 ± 0.6	0.047
Head length, cm	10.9 ± 0.2	12.2 ± 0.3	0.002
Heart weight, g	8.29 ± 0.52	8.92 ± 0.28	0.302
Liver weight, g	31.79 ± 2.15	28.51 ± 1.73	0.255
Spleen weight, g	0.98 ± 0.05	1.10 ± 0.05	0.095
Kidney weight, g	8.74 ± 0.55	8.66 ± 0.63	0.924
Stomach weight, g	5.63 ± 0.35	6.60 ± 0.65	0.207
Pancreas weight, g	1.41 ± 0.11	1.50 ± 0.08	0.569
Large intestinal weight, g	12.45 ± 1.66	14.20 ± 1.26	0.414
Small intestinal weight, g	33.07 ± 2.75	36.85 ± 2.69	0.343
Small intestinal length, cm	286.8 ± 11.7	291.8 ± 3.3	0.725

Data are presented as means ± SEM (*n* = 8). CON, control group; Fiber, fiber group. Large intestine includes cecum, colon, and rectum; Small intestine includes duodenum, jejunum, and ileum.

**Table 3:** Plasma biochemical index of newborn piglets.

	CON	Fiber	*p*-Value
ALT, U/L	14.38 ± 2.77	14.00 ± 1.95	0.913
AST, U/L	36.87 ± 9.05	28.50 ± 3.19	0.398
GGT, U/L	42.00 ± 7.73	51.50 ± 6.30	0.357
TBA, µmol/L	4.95 ± 0.78	14.83 ± 6.98	0.182
GLU, mmol/L	2.78 ± 0.16	2.79 ± 0.21	0.961
Urea, mmol/L	2.97 ± 0.23	3.01 ± 0.12	0.884
CREA, mmol/L	72.45 ± 4.25	89.60 ± 9.95	0.003
TG, mmol/L	0.15 ± 0.02	0.12 ± 0.01	0.156

Data are presented as means ± SEM (*n* = 8). CON, control group; Fiber, fiber group. ALT, alanine aminotransferase; AST, aspartate aminotransferase; GGT, gamma-glutamyltransferase; TBA, total bile acid; GLU, glucose; CREA, creatinine; TG, triglyceride.

**Table 4:** The relative abundance of main bacterial phyla in the colon of newborn piglets.

	CON	Fiber	*p*-Value
Proteobacteria	63.91 ± 8.13	43.25 ± 7.53	0.132
Firmicutes	23.74 ± 8.65	30.52 ± 7.99	0.589
Bacteroidetes	4.04 ± 1.22	11.85 ± 3.71	0.065
Actinobacteria	1.56 ± 0.77	4.69 ± 1.91	0.132
Cyanobacteria	4.30 ± 2.02	3.78 ± 2.29	0.818
Fusobacteria	0.21 ± 0.06	0.87 ± 0.56	0.485
Tenericutes	0.17 ± 0.10	0.14 ± 0.10	0.699
Acidobacteria	0.04 ± 0.02	0.85 ± 0.37	0.015
Chloroflexi	0.12 ± 0.07	0.60 ± 0.37	0.180
unidentified_Bacteria	0.12 ± 0.03	0.74 ± 0.35	0.065
Others	1.78 ± 0.60	2.71 ± 0.48	0.310

Data are presented as means ± SEM (*n* = 6). CON, control group; Fiber, fiber group.

**Table 5:** Biochemical information about proteins differentially expressed in the ileum of newborn piglets.

Accession	Description	Gene Name	Protein Name	Fold Change	*p*-Value
**Cellular apoptosis**					
F4ZS19	Phospholipid scramblase OS = Sus scrofa OX = 9823 GN = PLSCR1 PE = 2 SV = 1 − [F4ZS19_PIG]	PLSCR1	Phospholipid scramblase	−0.81	0.0393
F1RR64	Endonuclease G OS = Sus scrofa OX = 9823 GN = ENDOG PE = 1 SV=1 − [F1RR64_PIG]	ENDOG	Endonuclease G	−0.63	0.0102
F1S0V3	Annexin OS = Sus scrofa OX = 9823 GN = ANXA6 PE = 1 SV=3 − [F1S0V3_PIG]	ANXA6	Annexin	−0.54	0.0472
A0A0B8RTB1	Apoptosis antagonizing transcription factor OS = Sus scrofa domesticus OX = 9825 GN = AATF PE = 4 SV = 1 − [A0A0B8RTB1_PIG]	AATF	Apoptosis antagonizing transcription factor	−0.82	0.0436
F1RKI3	Histidine triad nucleotide binding protein 1 OS = Sus scrofa OX = 9823 GN = HINT1 PE = 1 SV = 1 − [F1RKI3_PIG]	HINT1	Histidine triad nucleotide binding protein 1	−0.71	0.0291
**Oxidative status**					
I3LQ80	Vitamin K epoxide reductase complex, subunit 1-like 1 OS = Sus scrofa OX = 9823 GN = VKORC1L1 PE = 1 SV = 2 − [I3LQ80_PIG]	VKORC1L1	Vitamin K epoxide reductase complex, subunit 1-like 1	+1.26	0.0230
F1S2N0	Glutathione S-transferase zeta 1 OS = Sus scrofa OX = 9823 GN = GSTZ1 PE = 1 SV = 2 − [F1S2N0_PIG]	GSTZ1	Glutathione S-transferase zeta 1	−0.82	0.0355
**Energy metabolism**					
Q29265	Phosphoenolpyruvate carboxykinase, cytosolic (Fragment) OS = Sus scrofa OX = 9823 PE = 2 SV = 1 − [Q29265_PIG]	PEPCK	Phosphoenolpyruvate carboxykinase, cytosolic (Fragment)	+1.35	0.0061
K9IVI1	2-oxoglutarate dehydrogenase, mitochondrial OS = Sus scrofa OX = 9823 GN = OGDH PE = 1 SV = 1 − [K9IVI1_PIG]	OGDH	2-oxoglutarate dehydrogenase, mitochondrial	+1.57	0.0346
F1RJ12	Protein phosphatase 1 regulatory subunit OS = Sus scrofa OX = 9823 GN = PPP1R3D PE = 4 SV = 1 − [F1RJ12_PIG]	PPP1R3D	Protein phosphatase 1 regulatory subunit	+1.21	0.0201
Q2EN80	NADH dehydrogenase 1 beta subcomplex 6 OS = Sus scrofa OX = 9823 GN = NDUFB6 PE = 1 SV = 1 − [Q2EN80_PIG]	NDUFB6	NADH dehydrogenase 1 beta subcomplex 6	+1.21	0.0034
F1S1J5	Glucose-6-phosphatase OS = Sus scrofa OX = 9823 GN = G6PC3 PE = 3 SV = 1 − [F1S1J5_PIG]	G6PC3	Glucose-6-phosphatase	−0.80	0.0109
F1RQQ8	Alpha-1,4 glucan phosphorylase OS = Sus scrofa OX = 9823 GN = PYGM PE = 1 SV = 2 − [F1RQQ8_PIG]	PYGM	Alpha-1,4 glucan phosphorylase	−0.77	0.0380
G3CKJ2	Glyceraldehyde-3-phosphate dehydrogenase (Fragment) OS = Sus scrofa OX = 9823 GN = GAPDH PE = 2 SV = 1 − [G3CKJ2_PIG]	GAPDH	Glyceraldehyde-3-phosphate dehydrogenase (Fragment)	−0.76	0.0030
**Immune and inflammatory responses**					
I3LEA8	Unc-93 homolog B1, TLR signaling regulator OS = Sus scrofa OX = 9823 GN = UNC93B1 PE = 4 SV = 2 − [I3LEA8_PIG]	UNC93B1	Unc-93 homolog B1, TLR signaling regulator	+1.83	0.0264
A0A287AP27	Uncharacterized protein OS = Sus scrofa OX = 9823 GN = CDK9 PE = 4 SV = 1 − [A0A287AP27_PIG]	CDK9	none	+1.40	0.0079
Q2TJZ7	Microsomal prostaglandin E synthase-1 OS = Sus scrofa OX = 9823 GN = PTGES PE = 1 SV = 1 − [Q2TJZ7_PIG]	PTGES	Microsomal prostaglandin E synthase-1	+1.39	0.0259
Q5W7K7	Interleukin 6 (Fragment) OS = Sus scrofa OX = 9823 GN = IL-6 PE = 2 SV = 1 − [Q5W7K7_PIG]	IL-6	Interleukin 6 (Fragment)	+1.26	0.0128
F1S9T2	Uncharacterized protein OS = Sus scrofa OX = 9823 GN = IFI44 PE = 1 SV = 2 − [F1S9T2_PIG]	IFI44	none	+1.24	0.0122
F1RIC1	Coiled-coil domain containing 86 OS = Sus scrofa OX = 9823 GN = CCDC86 PE = 4 SV = 1 − [F1RIC1_PIG]	CCDC86	Coiled-coil domain containing 86	−0.80	0.0107
F1RTQ6	Histone H2B OS = Sus scrofa OX = 9823 GN = HIST1H2BA PE = 1 SV = 1 − [F1RTQ6_PIG]	HIST1H2BA	Histone H2B	−0.80	0.0162
**Cell structure and motility**					
F1RFA8	MTOR associated protein, LST8 homolog OS = Sus scrofa OX = 9823 GN = MLST8 PE = 1 SV = 3− [F1RFA8_PIG]	MLST8	MTOR associated protein, LST8 homolog	+1.51	0.0162
F2Z5K5	Tubulin beta chain OS = Sus scrofa OX = 9823 GN = TUBB4A PE = 1 SV = 2 − [F2Z5K5_PIG]	TUBB4A	Tubulin beta chain	−0.80	0.0264
K9IVQ6	Plectin (Fragment) OS = Sus scrofa OX = 9823 GN = PLEC PE = 2 SV = 1 − [K9IVQ6_PIG]	PLEC	Plectin (Fragment)	−0.73	0.0451
A0A287AHG5	Protein kinase C and casein kinase substrate in neurons 1 OS = Sus scrofa OX = 9823 GN = PACSIN1 PE = 1 SV = 1 − [A0A287AHG5_PIG]	PACSIN1	Protein kinase C and casein kinase substrate in neurons 1	−0.68	0.0083
A0A287AYM2	Glial fibrillary acidic protein OS = Sus scrofa OX = 9823 GN = GFAP PE = 1 SV = 1 − [A0A287AYM2_PIG]	GFAP	Glial fibrillary acidic protein	−0.56	0.0146

Compared with control group, + means this protein was up-regulated in fiber group; − means this protein was down-regulated in fiber group; none means no gene name or protein name for this protein.

## Data Availability

The data presented in this study are available in article or supplementary material.

## Supplementary Materials

The following are available online at https://www.mdpi.com/2072-6643/13/1/42/s1, Additional file 1: Table S1. Primer sequences of the target and reference genes. Table S2. The relative abundance of main bacterial genus in the colon of newborn piglets. Figure S1. Comparison of the compositions of the colon microbiota between two groups by Principal Co-ordinates analysis (PCoA). Additional file 2: Table S3. Proteins identification list. Table S4. Differentially abundant proteins list.

## References

[1] Takiishi T., Fenero C.I.M., Camara N.O.S. (2017). Intestinal barrier and gut microbiota: Shaping our immune responses throughout life. Tissue Barriers.

[2] Guilloteau P., Zabielski R., Hammon H.M., Metges C.C. (2010). Nutritional programming of gastrointestinal tract development. Is the pig a good model for man?. Nutr. Res. Rev..

[3] Peng X., Yan C., Hu L., Liu Y., Xu Q., Wang R., Qin L., Wu C., Fang Z., Lin Y. (2019). Effects of Fat Supplementation during Gestation on Reproductive Performance, Milk Composition of Sows and Intestinal Development of their Offspring. Animals.

[4] Azad M.A.K., Bin P., Liu G., Fang J., Li T., Yin Y. (2018). Effects of different methionine levels on offspring piglets during late gestation and lactation. Food Funct..

[5] Reyes-Camacho D., Vinyeta E., Perez J.F., Aumiller T., Criado L., Palade L.M., Taranu I., Folch J.M., Calvo M.A., Van der Klis J.D. (2020). Phytogenic actives supplemented in hyperprolific sows: Effects on maternal transfer of phytogenic compounds, colostrum and milk features, performance and antioxidant status of sows and their offspring, and piglet intestinal gene expression. J. Anim. Sci..

[6] Wang W., Chen D., Yu B., Huang Z., Mao X., Zheng P., Luo Y., Yu J., Luo J., Yan H. (2020). Effects of dietary inulin supplementation on growth performance, intestinal barrier integrity and microbial populations in weaned pigs. Br. J. Nutr..

[7] Carlson J.L., Erickson J.M., Lloyd B.B., Slavin J.L. (2018). Health Effects and Sources of Prebiotic Dietary Fiber. Curr. Dev. Nutr..

[8] Koh A., De Vadder F., Kovatcheva-Datchary P., Backhed F. (2016). From Dietary Fiber to Host Physiology: Short-Chain Fatty Acids as Key Bacterial Metabolites. Cell.

[9] Makki K., Deehan E.C., Walter J., Backhed F. (2018). The Impact of Dietary Fiber on Gut Microbiota in Host Health and Disease. Cell Host Microbe.

[10] Couto M.R., Goncalves P., Magro F., Martel F. (2020). Microbiota-derived butyrate regulates intestinal inflammation: Focus on inflammatory bowel disease. Pharm. Res..

[11] Lin S.M., Zhou X.M., Zhou Y.L., Kuang W.M., Chen Y.J., Luo L., Dai F.Y. (2020). Intestinal morphology, immunity and microbiota response to dietary fibers in largemouth bass, Micropterus salmoide. Fish. Shellfish Immunol..

[12] Chen T., Chen D., Tian G., Zheng P., Mao X., Yu J., He J., Huang Z., Luo Y., Luo J. (2020). Effects of soluble and insoluble dietary fiber supplementation on growth performance, nutrient digestibility, intestinal microbe and barrier function in weaning piglet. Anim. Feed Sci. Technol..

[13] Yu L., Zhong X., He Y., Shi Y. (2020). Butyrate, but not propionate, reverses maternal diet-induced neurocognitive deficits in offspring. Pharm. Res..

[14] Nakajima A., Kaga N., Nakanishi Y., Ohno H., Miyamoto J., Kimura I., Hori S., Sasaki T., Hiramatsu K., Okumura K. (2017). Maternal High Fiber Diet during Pregnancy and Lactation Influences Regulatory T Cell Differentiation in Offspring in Mice. J. Immunol..

[15] Lin Y., Li L., Li Y., Wang K., Wei D., Xu S., Feng B., Che L., Fang Z., Li J. (2019). Interpretation of Fiber Supplementation on Offspring Testicular Development in a Pregnant Sow Model from a Proteomics Perspective. Int. J. Mol. Sci..

[16] Li Y., Liu H., Zhang L., Yang Y., Lin Y., Zhuo Y., Fang Z., Che L., Feng B., Xu S. (2019). Maternal Dietary Fiber Composition during Gestation Induces Changes in Offspring Antioxidative Capacity, Inflammatory Response, and Gut Microbiota in a Sow Model. Int. J. Mol. Sci..

[17] Livak K.J., Schmittgen T.D. (2001). Analysis of relative gene expression data using real-time quantitative PCR and the 2(-Delta Delta C(T)) Method. Methods.

[18] Wisniewski J.R., Zougman A., Nagaraj N., Mann M. (2009). Universal sample preparation method for proteome analysis. Nat. Methods.

[19] Hansen C.F., Hales J., Amdi C., Moustsen V.A. (2019). Intrauterine growth-restricted piglets defined by their head shape have impaired survival and growth during the suckling period. Anim. Prod. Sci..

[20] Douglas S.L., Edwards S.A., Kyriazakis I. (2016). Are all piglets born lightweight alike? Morphological measurements as predictors of postnatal performance. J. Anim. Sci..

[21] Ding X., Li H., Wen Z., Hou Y., Wang G., Fan J., Qian L. (2020). Effects of Fermented Tea Residue on Fattening Performance, Meat Quality, Digestive Performance, Serum Antioxidant Capacity, and Intestinal Morphology in Fatteners. Animals.

[22] Meyer A.M., Caton J.S. (2016). Role of the Small Intestine in Developmental Programming: Impact of Maternal Nutrition on the Dam and Offspring. Adv. Nutr..

[23] Yao Y., Voillet V., Jegou M., SanCristobal M., Dou S., Rome V., Lippi Y., Billon Y., Pere M.C., Boudry G. (2017). Comparing the intestinal transcriptome of Meishan and Large White piglets during late fetal development reveals genes involved in glucose and lipid metabolism and immunity as valuable clues of intestinal maturity. BMC Genom..

[24] Goulet O. (2015). Potential role of the intestinal microbiota in programming health and disease. Nutr. Rev..

[25] Cheng C., Wei H., Xu C., Xie X., Jiang S., Peng J. (2018). Maternal Soluble Fiber Diet during Pregnancy Changes the Intestinal Microbiota, Improves Growth Performance, and Reduces Intestinal Permeability in Piglets. Appl. Env. Microbiol..

[26] Xie J.-J., Liu Q.-q., Liao S., Fang H.-H., Yin P., Xie S.-W., Tian L.-X., Liu Y.-J., Niu J. (2019). Effects of dietary mixed probiotics on growth, non-specific immunity, intestinal morphology and microbiota of juvenile pacific white shrimp, *Litopenaeus vannamei*. Fish. Shellfish Immunol..

[27] Konstantinov S.R., Favier C.F., Zhu W.Y., Williams B.A., Klüß J., Souffrant W.-B., de Vos W.M., Akkermans A.D.L., Smidt H. (2004). Microbial diversity studies of the porcine gastrointestinal ecosystem during weaning transition. Anim. Res..

[28] Turnbaugh P.J., Ley R.E., Mahowald M.A., Magrini V., Mardis E.R., Gordon J.I. (2006). An obesity-associated gut microbiome with increased capacity for energy harvest. Nature.

[29] Kielak A.M., Barreto C.C., Kowalchuk G.A., Van V.J.A., Kuramae E.E. (2016). The Ecology of Acidobacteria: Moving beyond Genes and Genomes. Front. Microbiol..

[30] Zheng X., Duan Y., Dong H., Zhang J. (2020). The effect of Lactobacillus plantarum administration on the intestinal microbiota of whiteleg shrimp Penaeus vannamei. Aquaculture.

[31] Ferrario C., Statello R., Carnevali L., Mancabelli L., Milani C., Mangifesta M., Duranti S., Lugli G.A., Jimenez B., Lodge S. (2017). How to Feed the Mammalian Gut Microbiota: Bacterial and Metabolic Modulation by Dietary Fibers. Front. Microbiol..

[32] Kong X.F., Ji Y.J., Li H.W., Zhu Q., Blachier F., Geng M.M., Chen W., Yin Y.L. (2016). Colonic luminal microbiota and bacterial metabolite composition in pregnant Huanjiang mini-pigs: Effects of food composition at different times of pregnancy. Sci. Rep..

[33] Nakajima A., Sasaki T., Itoh K., Kitahara T., Takema Y., Hiramatsu K., Ishikawa D., Shibuya T., Kobayashi O., Osada T. (2020). A Soluble Fiber Diet Increases Bacteroides fragilis Group Abundance and Immunoglobulin A Production in the Gut. Appl. Environ. Microbiol..

[34] Singh A., Mittal M. (2019). Neonatal microbiome—A brief review. J. Matern. Neonatal Med..

[35] Walker R.W., Clemente J.C., Peter I., Loos R.J.F. (2017). The prenatal gut microbiome: Are we colonized with bacteria in utero?. Pediatr. Obes..

[36] Thorburn A.N., McKenzie C.I., Shen S., Stanley D., Macia L., Mason L.J., Roberts L.K., Wong C.H., Shim R., Robert R. (2015). Evidence that asthma is a developmental origin disease influenced by maternal diet and bacterial metabolites. Nat. Commun..

[37] Hu M., Eviston D., Hsu P., Marino E., Chidgey A., Santner-Nanan B., Wong K., Richards J.L., Yap Y.A., Collier F. (2019). Decreased maternal serum acetate and impaired fetal thymic and regulatory T cell development in preeclampsia. Nat. Commun..

[38] Wang X., Lin G., Liu C., Feng C., Zhou H., Wang T., Li D., Wu G., Wang J. (2014). Temporal proteomic analysis reveals defects in small-intestinal development of porcine fetuses with intrauterine growth restriction. J. Nutr. Biochem..

[39] Suarez-Trujillo A., Chen Y., Aduwari C., Cummings S., Kuang S., Buhman K.K., Hedrick V., Sobreira T.J.P., Aryal U.K., Plaut K. (2019). Maternal high-fat diet exposure during gestation, lactation, or gestation and lactation differentially affects intestinal morphology and proteome of neonatal mice. Nutr. Res..

[40] Mao X., Hu H., Xiao X., Chen D., Yu B., He J., Yu J., Zheng P., Luo J., Luo Y. (2019). Lentinan administration relieves gut barrier dysfunction induced by rotavirus in a weaned piglet model. Food Funct..

[41] Sivagnanam U., Palanirajan S.K., Gummadi S.N. (2017). The role of human phospholipid scramblases in apoptosis: An overview. Biochim. Biophys. Acta Mol. Cell Res..

[42] Azami M., Ranjkesh Adermanabadi V., Khanahmad H., Mohaghegh M.A., Zaherinejad E., Aghaei M., Jalali A., Hejazi S.H. (2018). Immunol.ogy and Genetic of Leishmania infantum: The Role of Endonuclease G in the Apoptosis. J. Res. Med. Sci..

[43] Tan Y., Wang Q., Zhao B., She Y., Bi X. (2016). GNB2 is a mediator of lidocaine-induced apoptosis in rat pheochromocytoma PC12 cells. Neurotoxicology.

[44] Weiske J., Huber O. (2006). The histidine triad protein Hint1 triggers apoptosis independent of its enzymatic activity. J. Biol. Chem..

[45] Liang X., Ni X., Wang Y., Xie J., Zhang X., Gu H., Zhang J. (2017). Jinhong Tablet Reduces Damage of Int.estinal Mucosal Barrier in Rats with Acute Biliary Infection via Bcl-2/Bax mRNA and Protein Regulation. Evid. Based Complement. Altern. Med..

[46] Westhofen P., Watzka M., Marinova M., Hass M., Kirfel G., Muller J., Bevans C.G., Muller C.R., Oldenburg J. (2011). Human vitamin K 2,3-epoxide reductase complex subunit 1-like 1 (VKORC1L1) mediates vitamin K-dependent intracellular antioxidant function. J. Biol. Chem..

[47] Westhofen P., Watzka M., Marinova M., Hass M., Oldenburg J. (2010). New insight into the function of VKORC1L1. Hamostaseologie.

[48] Ma J.W., Ji D.D., Li Q.Q., Zhang T., Luo L. (2020). Inhibition of connexin 43 attenuates oxidative stress and apoptosis in human umbilical vein endothelial cells. BMC Pulm. Med..

[49] Wang M., Huang H., Liu S., Zhuang Y., Yang H., Li Y., Chen S., Wang L., Yin L., Yao Y. (2019). Tannic acid modulates intestinal barrier functions associated with intestinal morphology, antioxidative activity, and intestinal tight junction in a diquat-induced mouse model. RSC Adv..

[50] Wen C., Guo Q., Wang W., Duan Y., Zhang L., Li J., He S., Chen W., Li F. (2020). Taurine Alleviates Int.estinal Injury by Mediating Tight Junction Barriers in Diquat-Challenged Piglet Models. Front. Physiol..

[51] Van der Beek C.M., Canfora E.E., Kip A.M., Gorissen S.H.M., Olde Damink S.W.M., van Eijk H.M., Holst J.J., Blaak E.E., Dejong C.H.C., Lenaerts K. (2018). The prebiotic inulin improves substrate metabolism and promotes short-chain fatty acid production in overweight to obese men. Metabolism.

[52] Rabot S., Membrez M., Blancher F., Berger B., Moine D., Krause L., Bibiloni R., Bruneau A., Gerard P., Siddharth J. (2016). High fat diet drives obesity regardless the composition of gut microbiota in mice. Sci. Rep..

[53] Most J., Goossens G.H., Reijnders D., Canfora E.E., Penders J., Blaak E.E. (2017). Gut microbiota composition strongly correlates to peripheral insulin sensitivity in obese men but not in women. Benef. Microbes.

[54] Boby R.G., Leelamma S. (2003). Blackgram fiber (*Phaseolus mungo*): Mechanism of hypoglycemic action. Plant. Foods Hum. Nutr..

[55] Choi J.S., Kim H., Jung M.H., Hong S., Song J. (2010). Consumption of barley beta-glucan ameliorates fatty liver and insulin resistance in mice fed a high-fat diet. Mol. Nutr. Food Res..

[56] Yoshida H., Ishii M., Akagawa M. (2019). Propionate suppresses hepatic gluconeogenesis via GPR43/AMPK signaling pathway. Arch. Biochem. Biophys..

[57] Rubio-Villena C., Sanz P., Garcia-Gimeno M.A. (2015). Structure-Function Analysis of PPP1R3D, a Protein Phosphatase 1 Targeting Subunit, Reveals a Binding Motif for 14-3-3 Proteins which Regulates its Glycogenic Properties. PLoS ONE.

[58] Oligschlaeger Y., Miglianico M., Dahlmans V., Rubio-Villena C., Chanda D., Garcia-Gimeno M.A., Coumans W.A., Liu Y., Voncken J.W., Luiken J.J. (2016). The interaction between AMPKbeta2 and the PP1-targeting subunit R6 is dynamically regulated by intracellular glycogen content. Biochem. J..

[59] Mithieux G., Andreelli F., Magnan C. (2009). Intestinal gluconeogenesis: Key signal of central control of energy and glucose homeostasis. Curr. Opin. Clin. Nutr. Metab. Care.

[60] Loublier S., Bayot A., Rak M., El-Khoury R., Benit P., Rustin P. (2011). The NDUFB6 subunit of the mitochondrial respiratory chain complex I is required for electron transfer activity: A proof of principle study on stable and controlled RNA interference in human cell lines. Biochem. Biophys. Res. Commun..

[61] Hoshino A., Fujii H. (2007). Redundant promoter elements mediate IL-3-induced expression of a novel cytokine-inducible gene, cyclon. FEBS Lett..

[62] Chen R., Kang R., Fan X.G., Tang D. (2014). Release and activity of histone in diseases. Cell Death Dis..

[63] Pelka K., Bertheloot D., Reimer E., Phulphagar K., Schmidt S.V., Christ A., Stahl R., Watson N., Miyake K., Hacohen N. (2018). The Chaperone UNC93B1 Regulates Toll-like Receptor Stability Independently of Endosomal TLR Transport. Immunity.

[64] Casrouge A., Zhang S.Y., Eidenschenk C., Jouanguy E., Puel A., Yang K., Alcais A., Picard C., Mahfoufi N., Nicolas N. (2006). Herpes simplex virus encephalitis in human UNC-93B deficiency. Science.

[65] Ross J.A., Vissers J.P.C., Nanda J., Stewart G.D., Husi H., Habib F.K., Hammond D.E., Gethings L.A. (2020). The influence of hypoxia on the prostate cancer proteome. Clin. Chem. Lab. Med..

[66] Bouameur J.E., Favre B., Borradori L. (2014). Plakins, a versatile family of cytolinkers: Roles in skin integrity and in human diseases. J. Investig. Derm..

[67] Karashima T., Tsuruta D., Hamada T., Ishii N., Ono F., Hashikawa K., Ohyama B., Natsuaki Y., Fukuda S., Koga H. (2012). Int.eraction of plectin and intermediate filaments. J. Derm. Sci..

[68] Bai X., Zheng X. (2013). Tip-to-tip interaction in the crystal packing of PACSIN 2 is important in regulating tubulation activity. Protein Cell.

[69] Li D., Liu X., Liu T., Liu H., Tong L., Jia S., Wang Y.F. (2020). Neurochemical regulation of the expression and function of glial fibrillary acidic protein in astrocytes. Glia.

[70] Jacinto E., Loewith R., Schmidt A., Lin S., Ruegg M.A., Hall A., Hall M.N. (2004). Mammalian TOR complex 2 controls the actin cytoskeleton and is rapamycin insensitive. Nat. Cell Biol..

[71] Pan F., Xu X., Zhang L.L., Luo H.J., Chen Y., Long L., Wang X., Zhuang P.T., Li E.M., Xu L.Y. (2020). Dietary riboflavin deficiency induces genomic instability of esophageal squamous cells that is associated with gut microbiota dysbiosis in rats. Food Funct..

[72] Tarnopolsky M.A. (2018). Myopathies Related to Glycogen Metabolism Disorders. Neurotherapeutics.

