# Supplementary material for: Effects of Maternal Fiber Intake on Intestinal Morphology, Bacterial Profile and Proteome of Newborns Using Pig as Model
Source: Nutrients. 2020 Dec 25;13(1):42. doi: 10.3390/nu13010042 (PMC7823571; doi:10.3390/nu13010042)
Supplement: Supplementary file 1 [file nutrients-13-00042-s001.zip › supplementary/supplementary materials-Additional file 1 - R2.docx]

**Supplementary materials**

**Table S1.** Primer sequences of the target and reference genes.

| **Genes** | **Primer sequence (5’ –3’)** | **Product (bp)** | **GenBank accession** |
| --- | --- | --- | --- |
| *ENDOG* | F: TGCGCGGCTCCTTTAAGAACT | 101 | XM_003122208.4 |
|  | R: GTGGATTCTGGCCTGAGCTG |  |  |
| *VKORC1L1* | F: ATCCTGCTCTCCATCTACGCCTAC | 137 | XM_003124445.4 |
|  | R: AGACCAAATCCTCGACCCCATCTG |  |  |
| *PEPCK* | F: GGCGTGTACTGGGAAGGCATTG | 126 | NM_001161753.1 |
|  | R: GGCACAGAAGCGAGAGTTAGGATG |  |  |
| *PPP1R3D* | F: CTGGCGCAGGTCAAGGTGTTC | 93 | XM_003134506.4 |
|  | R: GCAGCACAGGTCCGAGTTGATG |  |  |
| *OGDH* | F: GGGGACACCGAGGGGAAGAAG | 113 | XM_021079061.1 |
|  | R: CGTGGACAGTGCCGTGAGTTG |  |  |
| *UNC93B1* | F: ACCTACCGCGAGGTGAAGTA | 199 | XM_003122436.6 |
|  | R: AGTAGCGCTCCCAGTAGTTG |  |  |
| *IL6* | F: TGCAGTCACAGAACGAGTGG | 116 | NM_214399.1 |
|  | R: CAGGTGCCCCAGCTACATTAT |  |  |
| *CCDC86* | F: AAGGAGCGGCAGGAGAGGAAG | 118 | XM_003122668.4 |
|  | R: CCAGGCGGCGTTTCAGGTTC |  |  |
| *GFAP* | F: CGACCAACTCACCGCCAACG | 122 | NM_001244397.1 |
|  | R: TGTTCTCAGCCTCCAGCCTCAG |  |  |
| *PACSIN1* | F: CAAGGAGGTGCTGCTGGACATC | 135 | XM_021098608.1 |
|  | R: GTGCTGCGGAACCATCTGAGATC |  |  |
| *TUBB4A* | F: TGACTCTCCTGGGACTACCAC | 88 | XM_003480812.4 |
|  | R: GGTTCAAGGGGGTTGGAGAT |  |  |
| *GAPDH* | F: CAAGGCTGTGGGCAAGGTCATC | 111 | NM_001206359.1 |
|  | R: TTCTCCAGGCGGCAGGTCAG |  |  |

ENDOG, endonuclease G; VKORC1L1, vitamin K epoxide reductase complex subunit 1 like 1; PEPCK, phosphoenolpyruvate carboxykinase 2; PPP1R3D, protein phosphatase 1 regulatory subunit 3D; OGDH, oxoglutarate dehydrogenase; UNC93B1, unc-93 homolog B1; IL6, interleukin 6; CCDC86, coiled-coil domain containing 86; GFAP, glial fibrillary acidic protein; PACSIN1, protein kinase C and casein kinase substrate in neurons 1; TUBB4A, tubulin beta 4A class Iva.

**Table S2.** The relative abundance of main bacterial genus in the colon of newborn piglets

|  | **CON** | **Fiber** | ***P*-value** |
| --- | --- | --- | --- |
| *Sphingomonas* | 42.21±13.32 | 5.96±4.16 | 0.093 |
| *unidentified_Clostridiales* | 1.63±0.60 | 1.41±0.60 | 0.485 |
| *Bradyrhizobium* | 1.75±0.65 | 9.54±5.18 | 0.041 |
| *Sphingobacterium* | 0.16±0.15 | 3.95±3.90 | 0.937 |
| *Pseudomonas* | 0.34±0.22 | 3.99±3.55 | 0.065 |
| *Ignatzschineria* | 1.10±1.02 | 3.71±3.00 | 0.699 |
| *Stenotrophomonas* | 3.12±3.07 | 3.80±2.55 | 0.065 |
| *Weissella* | 2.73±2.71 | 0.08±0.07 | 0.240 |
| *unidentified_Cyanobacteria* | 4.44±1.95 | 3.75±2.29 | 0.589 |
| *Lactobacillus* | 1.05±0.35 | 3.02±2.28 | 0.699 |
| *Enterococcus* | 0.12±0.05 | 0.51±0.28 | 0.485 |
| *Streptococcus* | 1.02±0.36 | 2.65±2.17 | 0.589 |
| *Alloprevotella* | 0.23±0.09 | 2.09±1.85 | 0.485 |
| *Desulfovibrio* | 1.82±1.80 | 0.04±0.01 | 0.310 |
| *Bifidobacterium* | 0.81±0.64 | 1.88±1.68 | 0.310 |
| *unidentified_Enterobacteriaceae* | 0.06±0.03 | 0.12±0.10 | 0.937 |
| *Salegentibacter* | 1.35±1.34 | 0.01±0.01 | 0.699 |
| *Virgibacillus* | 1.31±1.30 | 0.00±0.00 | 0.394 |
| *Klebsiella* | 0.12±0.03 | 1.29±1.17 | 0.818 |
| *Moraxella* | 1.91±1.15 | 0.91±0.53 | 0.937 |
| *Turicibacter* | 0.28±0.17 | 0.50±0.40 | 0.394 |
| *Nocardioides* | 1.05±1.04 | 0.10±0.06 | 0.485 |
| *Gramella* | 0.92±0.92 | 0.004±0.004 | 0.937 |
| *Romboutsia* | 0.53±0.25 | 1.70±0.96 | 0.589 |
| *Vagococcus* | 0.09±0.04 | 1.37±0.88 | 0.394 |
| *Marinobacter* | 0.82±0.82 | 0.02±0.01 | 0.310 |
| *Phyllobacterium* | 0.02±0.01 | 0.83±0.72 | 0.015 |
| *Haemophilus* | 0.08±0.02 | 0.77±0.70 | 0.589 |
| *Citrobacter* | 0.14±0.06 | 0.91±0.66 | 0.485 |
| *Faecalibacterium* | 0.62±0.20 | 0.83±0.48 | 0.818 |
| *Others* | 28.21±6.17 | 44.26±6.93 | 0.132 |

Data are presented as means ± SEM (*n*=6).

CON, control group; Fiber, fiber group.


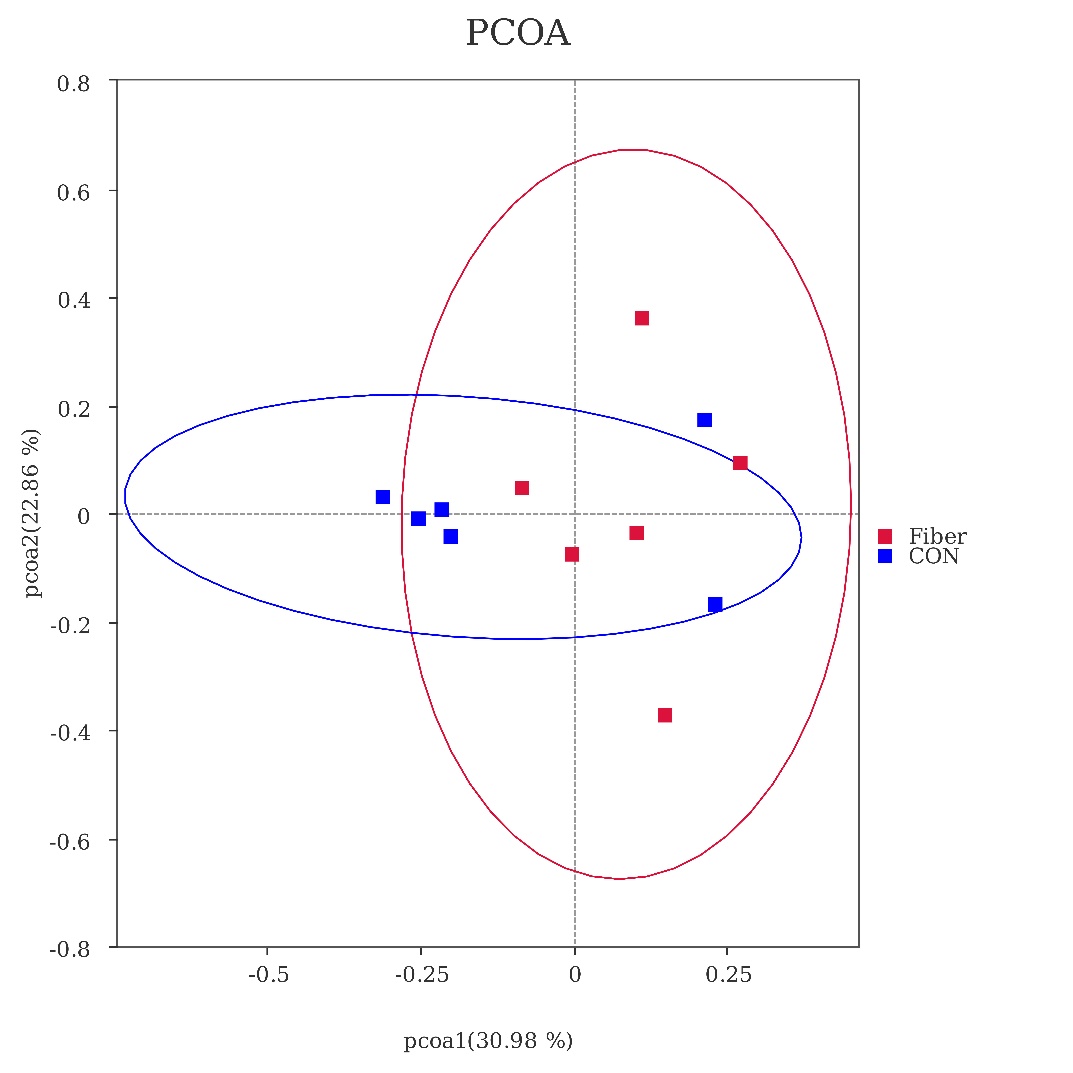


**Figure S1.** Comparison of the compositions of the colon microbiota between two groups by Principal Co-ordinates analysis (PCoA).
